# Birth after preeclamptic pregnancies: association with allergic sensitization and allergic rhinoconjunctivitis in late childhood; a historically matched cohort study

**DOI:** 10.1186/1471-2431-14-101

**Published:** 2014-04-11

**Authors:** Kristine Kjer Byberg, Bjorn Ogland, Geir Egil Eide, Knut Øymar

**Affiliations:** 1 Paediatric Department, Stavanger University Hospital, Post box 8100, N-4068 Stavanger, Norway; 2 Neonatal Intensive Care Unit, Oslo University Hospital Rikshospitalet, Oslo, Norway; 3 Centre for Clinical Research, Haukeland University Hospital, Bergen, Norway; 4 Department of Global Public Health and Primary Care, University of Bergen, Bergen, Norway; 5 Department of Clinical Medicine, University of Bergen, Bergen, Norway

**Keywords:** Childhood, Allergy, Allergic rhinoconjunctivitis, Allergic sensitization, Asthma, Atopic dermatitis, Atopy, Child, Lung function, Preeclampsia

## Abstract

**Background:**

The development of allergic sensitization and allergic disease may be related to factors during intrauterine life, but the role of maternal preeclampsia is not known.

We studied if maternal preeclampsia is associated with long-term allergic sensitization, allergic rhinoconjunctivitis, atopic dermatitis, asthma and with altered lung function in late childhood.

**Methods:**

617 children participated in a 1:2 matched and controlled historical cohort study; 230 born after preeclamptic pregnancies and 387 born after normotensive pregnancies. Specific IgE in serum and lung function were measured at the age of 12.8 years and questionnaires on maternal and adolescent data were completed at the ages of 10.8 years (girls) and 11.8 years (boys), and at 12.8 years (both genders). The association between birth after preeclampsia and the main outcome measures allergic sensitization, allergic rhinoconjunctivitis, atopic dermatitis, asthma and lung function in late childhood were analysed with multiple regression analyses, including possible confounders.

**Results:**

Severe maternal preeclampsia was associated with high level allergic sensitization (sum of specific IgE in serum ≥ 3.9 kU/l; the 25 percentile for all children being sensitized); odds ratio (OR): 3.79; 95% confidence interval (CI): (1.54, 9.32); p = 0.015 and with allergic rhinoconjunctivitis in offspring; OR: 2.22, 95% CI: (1.19, 4.14), p = 0.047. Preeclampsia was not associated with atopic dermatitis, asthma or altered lung function in late childhood.

**Conclusion:**

Maternal preeclampsia was associated with allergic sensitization and allergic rhinoconjunctivitis in offspring in late childhood, but not with other atopic diseases.

## Background

The prevalence of allergy and asthma has been increasing in both adults and children during the last decades [1]. There is also increasing evidence that early life events including intrauterine factors are important for the development of atopic disease [2-4].

Atopic diseases are associated with an inhibition of the transition towards an increased T-helper cell type 1 (Th1)/Th2 balance after birth, resulting in Th2 cytokine predominance [4,5]. Maternal inflammatory cytokines during pregnancy have been shown to correlate with corresponding cytokines in offspring at the age of one [6], and an association between an altered maternal cytokine profile and subsequent atopic disease in offspring has been suggested [7].

Preeclampsia is a common and potentially serious complication of the second half of pregnancy affecting both mother and child, characterised by maternal hypertension and proteinuria, and occasionally foetal growth restriction [8,9]. Preeclampsia is associated with an increase in several circulating maternal cytokines, and a skewed maternal immune response towards an increased Th1/Th2 balance [10]. This could potentially skew the child’s cytokine balance after birth in the same direction, and thereby protect against the development of atopic disease [6]. However, clinical studies have suggested that complications during pregnancy may rather increase the risk of childhood asthma and allergic rhinoconjunctivitis [11,12], and an association between maternal preeclampsia and subsequent allergic sensitization in offspring during adolescence has been suggested [13].

Preeclampsia has also been associated with an increased risk of RDS and BPD in preterm infants and to recurrent wheezing in a general population of pre-school children [14]. This association might either be due to an increased soluble antiangiogenic factor [15], or a congenital reduction in airways calibre and compliance in particular in infants with intrauterine growth restriction [16]. However, no studies have evaluated a possible long-term association between maternal preeclampsia and asthma or lung function in offspring.

In a long–term follow-up of children of preeclamptic and normal pregnancies, the aim was to study if maternal preeclampsia is associated with allergic sensitization, allergic rhinoconjunctivitis, atopic dermatitis, asthma, and lung function in late childhood.

## Methods

### Study population and design

**Figure 1:**
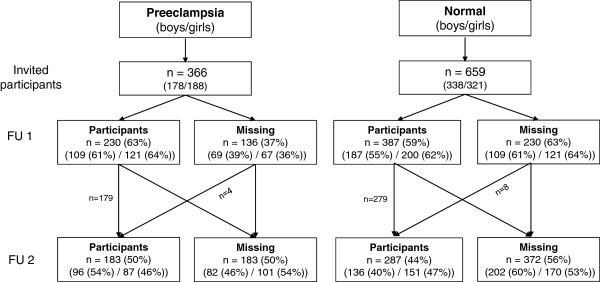
**Number of participants invited to the Stavanger study, and numbers that consented to first and second follow-up.***Abbreviations:* FU1 = First follow-up at the ages of 10.8 years (girls) and 11.8 years (boys); FU2 = Second follow-up at the age of 12.8 years (both genders); Preeclampsia = children born after pregnancies with preeclampsia; Normal = children born after pregnancies without preeclampsia.

The study was a part of “the Stavanger study” described in detail elsewhere [17]. In short, cord blood was drawn from all newborns at Stavanger University Hospital during 1993–1995, during which 12 804 deliveries took place. The Medical Birth Registry of Norway was used to identify offspring exposed to maternal preeclampsia and unexposed offspring, and information was verified and supplemented with data from hospital records. All offspring of preeclamptic pregnancies were defined as exposed. For each exposed, two matched unexposed offspring were selected as follows: one was defined as the next delivery in the hospital and one as the next delivery matched on maternal age. Exposed and unexposed offspring were invited to participate in a follow-up (FU) study at the target ages of 10.8 years (girls) and 11.8 years (boys) (FU1), and a second follow-up at the target age of 12.8 years (FU2) (Figure 1). The target ages at FU1 were selected to also be able to collect puberty stage data at an age presumed to represent the start of puberty development in the children [17]. If unexposed offspring did not respond, no substitutes were invited. Consequently, the study design was a historically matched cohort with 1025 children; 366 in the preeclampsia cohort and 659 in the control cohort.

The study was approved by the Norwegian Data Inspectorate, the Regional Committee for Ethics in medical research and the Institutional Review Board of the National Cancer Institute of the United States. Written consent was obtained from all participating children and mothers at follow-up.

### Data collection and definitions

Preeclampsia was defined as a diastolic blood pressure increased by ≥ 25 mmHg to a persistent pressure of ≥ 90 mmHg and proteinuria with dipstick ≥ +1 present in at least one urine sample after 20 weeks of gestation. Preeclampsia was further divided into mild, moderate and severe. Moderate preeclampsia was defined as proteinuria with dipstick ≥ +2. Severe preeclampsia was defined as proteinuria with dipstick ≥ + 3 and diastolic blood pressure of ≥110 mmHg.

Maternal body mass index (BMI, kg/m^2^) was calculated using weight measurement at the first antenatal visit at primary healthcare examination during the first trimester of pregnancy and height measurement from FU1. Maternal smoking in pregnancy was recorded at the same antenatal visit. Data on gestational age at birth and mode of delivery were extracted from hospital records. Birth weight for gestational age was calculated as z-scores based on Scandinavian normal standards [18]. Weight and height in offspring were recorded at FU1, and z-scores for BMI were calculated using the latest growth references for Norwegian children [19].

### Questionnaires

At FU1, the questions included the birth order of the child, parental asthma, and atopic disease of the child. The mothers were asked as follows: Has your child ever had asthma (diagnosed by physician), allergy in nose/eyes (hay fever) or atopic dermatitis (childhood eczema)?

At FU2, the children answered a questionnaire from the International Study of Asthma and Allergies in Childhood (ISAAC) translated into Norwegian [20]. Reported asthma symptoms and asthma medication during the last year and asthma diagnosis ever were recorded. Missing answers were interpreted as negative. Current asthma at FU2 was defined as asthma ever, in addition to asthma symptoms or the use of asthma medication during the last 12 months.

### Laboratory methods

At FU2 blood was drawn from the children, centrifuged and aliquoted, and serum stored at -80°C. Allergic sensitization was determined by serum specific immunoglobulin E (IgE) antibodies using Phadiatop^®^ and fx5E^®^ (ImmunoCAP^®^ 250, Phadia AB, Uppsala, Sweden). If Phadiatop^®^ was positive, serum was further analysed for specific IgE against *Dermatophagoides pteronyssinus*, cat, horse, dog, timothy, common silver birch, mugwort and *Cladosporium herbarum*. If fx5E^®^ was positive, serum was further analysed for specific IgE against egg white, milk, fish (cod), wheat, peanut and soya bean. Allergic sensitization was defined as specific IgE *≥* 0.35 *kU/l* for at least one allergen. The levels of specific IgE *≥* 0.35 were added together, and high level allergic sensitization was defined as a sum > 3.9 *kU/l*; the 25 percentile for all children being sensitized.

At FU2, lung function measures were performed by spirometry according to standard quality criteria [21] with a Vmax Encore spirometer (Sensor Medics Inc., Anaheim, USA).

### Statistics

Groups were compared with Pearson’s chi-square exact test for the dichotomous outcomes and independent t-tests (Gosset’s t-test) and one way analysis of variance for the continuous outcomes. Bonferroni-correction of p-values was applied to adjust for multiple testing.

Before analyses, the variable preeclampsia was categorised into none, mild/moderate (combined) and severe. Risk associations between preeclampsia and related pregnancy variables with outcomes in late childhood were analysed by multiple logistic and linear regression analyses, including the covariates gender, birthweight z-score for gestational age, being firstborn, maternal smoking during pregnancy, maternal age at birth, caesarean section, gestational age, maternal BMI and maternal asthma. Paternal asthma was not included as a covariate due to low response rate. Each variable was first entered separately into simple regression models. Covariates significant at the 10% level and those considered important were included in backward stepwise logistic and linear regression analyses. Final models included the remaining covariates significant at the 5% level and the covariates gender and maternal asthma, considered as biologically important. Analysing the ordinal variable preeclampsia as were it a continuous variable with scores 0, 1 and 2 for the three levels, ORs showing a trend have been calculated by multiple logistic regression analysis.

From logistic regression odds ratios (OR) with 95% confidence interval (CI) and likelihood ratio p-value (LR-p) for each exposure are reported. From linear regression the estimated coefficients (b), 95% CI and F-test p-value are reported. Interactions between preeclampsia and all other risk factors remaining in the final models were tested. Also interactions between preeclampsia and gender were tested.

All tests were 2-tailed and p-values ≤ 0.05 were considered statistically significant. Due to missing data, matching was not included in the analyses, but the matching variables were adjusted for. SPSS for Windows (version 18.0.0, Chicago, Ill., USA) was used for all analyses.

## Results

### Characteristics of the participants

The number of children invited and participating in FU1 and FU2 are shown in Figure 1. Table 1 shows the characteristics of children who consented to FU1, and those who did not consent. There were no significant differences in perinatal characteristics of children who consented and those who did not consent.

**Table 1:** **Initial characteristics of 1025 Norwegian children born in 1993–1995 and invited to the Stavanger study according to consenting or not to the first follow-up 11–12 years later**^**a)**^

	**Consented (n = 617)**			**Did not consent (n = 409)**			
**Variable**	**n**			**n**			**p**
Gender: boys, *n (%)*	617	296	(48.0)	408	220	(53.9)	0.065^b)^
Preeclampsia, *n (%)*	617	230	(37.3)	409	137	(33.5)	0.231^b)^
Maternal age, years, *mean, 95% CI*	610	28.0	(27.7, 28.4)	408	27.6	(27.2, 28.1)	0.194^c)^
Gestational age, weeks; *mean, 95% CI*	604	39.3	(39.1, 39.5)	343	39.2	(39.0, 39.5)	0.855^c)^
Birth weight z-score^d)^	590	-0.17	(-0.27, -0.07)	339	-0.06	(-0.18, 0.06)	0.169^c)^

*Abbreviations:CI* confidence interval.

^a)^Follow-up at the ages of 10.8 years (girls) and 11.8 years (boys);

^b)^Exact chi-square test;

^c)^Gosset’s t-test;

^d)^Number of standard deviations from mean.

At FU1, the age of the girls was 10.8 (±0.22) years (mean, SD), and for boys 11.8 (±0.18) years. At FU2 the age for both genders was 12.8 (±0.19) years.

BMI z-score at FU1 was higher in children who only consented to FU1 than in children who consented to both FU1 and FU2 (mean difference BMI z-score: 0.31 kg/m^2^; 95% CI: 0.13 to 0.49; p = 0.001). When analysed separately for girls and boys, the difference persisted for girls only (mean difference BMI: 0.44 kg/m^2^; 95% CI: 0.20 to 0.68; p < 0.001). More children had atopic dermatitis of those who only consented to FU1 (48/150; 32%), than those who consented to both FU1 and FU2 (100/445; 22.5%); p = 0.022. No other variables differed significantly between those who consented to FU1 only and those who consented to both FU1 and FU2.

### Preeclampsia and outcomes

The outcome in children according to maternal preeclampsia status is shown in Table 2. A greater proportion of offspring from pregnancies with severe preeclampsia had allergic rhinoconjunctivitis and high level allergic sensitization than offspring from pregnancies with mild/moderate or no preeclampsia.

**Table 2:** Atopy, asthma and lung function in late childhood in 586 Norwegian children according to mother’s preeclampsia status

	**Severe preeclampsia**			**Mild/moderate preeclampsia**			**No preeclampsia**			**Severe vs. no preeclampsia**	**Mild/moderate vs. no preeclampsia**	**Overall**
**Outcome variable**	**n**			**n**			**n**			**p-value**^**a)**^	**p-value**^**a)**^	**p-value**^**b)**^
**FU1 variables**												
Allergic rhinoconjunctivitis, *n* (%)	54	19	(35.2)	161	36	(22.4)	371	71	(19.1)	0.018	0.822	0.015
Atopic dermatitis, *n* (%)	54	15	(27.8)	163	37	(22.7)	369	92	(24.9)	1.476	1.172	1.000
Asthma, *n* (%)	53	9	(17.0)	161	14	(8.7)	366	30	(8.2)	0.092	1.730	0.100
**FU2 variables**												
Allergic sensitization, *n* (%)	39	15	(38.5)	112	40	(36.3)	230	72	(31.3)	0.918	0.924	0.296
High level allergic sensitization, *n* (%)^c)^	39	15	(38.5)	112	28	(25.0)	230	50	(21.7)	0.056	1.166	0.042
Current asthma, *n* (%)	46	6	(13.0)	123	12	(9.8)	279	19	(6.8)	0.454	0.630	0.126
FEV_1_%, *mean, 95% CI*	45	89.7	(87.0, 92.5)	126	91.0	(89.0, 93.0)	272	91.1	(90.0, 92.2)	1.000	1.000	0.682
FVC%, *mean, 95% CI*	45	99.2	(95.5, 102.8)	126	100.0	(97.7, 102.3)	272	101.1	(99.8, 102.4)	0.965	1.000	0.489
FEV_1_/FVC, *mean, 95% CI*	45	84.6	(82.7, 86.5)	126	85.3	(83.9, 86.6)	272	84.9	(84.1, 85.7)	1.000	1.000	0.482
FEF_25–75_%, *mean, 95% CI*	45	84.9	(79.4, 90.3)	126	89.6	(85.9, 93.4)	272	87.4	(85.1, 89.6)	1.000	0.827	0.317
FEF_25–75_/FVC, *mean, 95% CI*	45	54.5	(50.0, 59.0)	126	57.3	(54.7, 59.9)	272	55.7	(54.1, 57.4)	1.000	0.906	0.429

*Abbreviations:FU1* first follow-up at the ages of 10.8 years (girls) and 11.8 years (boys), *FU2* second follow-up at the age of 12.8 years (both genders), *FEV*_*1*_*%* forced expiratory volume in first second predicted, *CI* Confidence interval, *FVC%* forced vital capacity predicted, *FEF*_*25–75*_*%* forced expiratory flow between 25% and 75% of the forced vital capacity, predicted.

^a)^Pearson’s exact chi-square test (dichotomous variable) and one way analysis of variance (continuous variable) with Bonferroni corrections;

^b)^Cochran-Armitage test for dichotomous outcomes and one way analysis of variance for continuous outcomes;

^c)^Sum of specific IgE > 3.9 *kU/l.*

Among children with any allergic sensitization, 50% had symptoms of allergic rhinoconjunctivitis, whereas 5.6% of children with allergic rhinoconjunctivitis did not have any allergic sensitization.

In the unadjusted logistic regression analyses, severe preeclampsia was a risk factor for allergic rhinoconjunctivitis; OR: 2.29; 95% CI: 1.24 to 4.24; LR-p = 0.036. Table 3 shows the results of adjusted logistic regression analyses with different atopic diseases as outcomes according to maternal preeclampsia status. In fully adjusted analyses, severe preeclampsia was a significant risk factor for high level allergic sensitization, but not so for any other outcomes of atopic disease.

**Table 3:** Summary of logistic regression analyses of atopic diseases in late childhood in 586 Norwegian children according to mother’s preeclampsia status

**Effects of maternal preeclampsia**												
	**Fully adjusted**^**a)**^						**Final analysis**^**b)**^					
	**n**	**Mild/moderate preeclampsia**		**Severe preeclampsia**		**Likelihood-ratio-**	**n**	**Mild/moderate preeclampsia**		**Severe preeclampsia**		**Likelihood-ratio**
**Outcome variable**		**OR**	**95% CI**	**OR**	**95% CI**	**p**		**OR**	**95% CI**	**OR**	**95% CI**	**p**
**FU1 variables**												
Allergic rhinoconjunctivitis	514	1.21	(0.70, 2.07)	2.10	(0.86, 5.11)	0.268	586	1.25	(0.79, 1.97)	2.23	(1.20, 4.17)	0.046
Atopic dermatitis	513	0.90	(0.54, 1.50)	0.97	(0.39, 2.39)	0.914						____
Asthma	506	0.87	(0.38, 1.97)	0.72	(0.19, 2.77)	0.878						____
**FU2 variables**												
Allergic sensitization	329	1.49	(0.84, 2.63)	2.44	(0.93, 6.42)	0.138						____
High level allergic sensitization^c)^	329	1.64	(0.87, 3.11)	4.42	(1.58, 12.3)	0.015	347	1.60	(0.88, 2.91)	4.05	(1.62, 10.1)	0.010^d)^
Current asthma	388	1.11	(0.45, 2.73)	0.69	(0.15, 3.18)	0.802						____

*Abbreviations:OR* Odds ratio, *CI* Confidence interval, *FU1* first follow-up at the ages of 10.8 years (girls) and 11.8 years (boys), *FU2* second follow-up at the age of 12.8 years (both genders); Likelihood-ratio-p refers to exposure only.

^a)^Adjusted for gender, birth weight z-score adjusted for gestational age, being firstborn, Caesarean section, maternal smoking during pregnancy, gestational age in weeks, maternal age, maternal body mass index (kg/m^2^) and maternal asthma;

^b)^After backward stepwise selection from fully adjusted model with p ≤ 0.05; all final analyses include the covariates gender and maternal asthma as default.

^c)^High level allergic sensitization = Sum of specific IgE > 3.9 *kU/l;* the 25 percentile of sensitized children.

^d)^Adjusted for gender, maternal smoking during pregnancy, gestational age in weeks and maternal asthma. No significant interaction effects were found in final analyses.

In the backward stepwise regression analysis of high level allergic sensitization the final model included preeclampsia as a significant risk factor, in addition to male gender, maternal smoking during pregnancy, gestational age in weeks and maternal asthma (Table 3). Specifically, birth after severe preeclampsia gave 4.05 times higher odds for high level allergic sensitization than birth after non-preeclampsia, adjusted for the other variables.

In a backward stepwise regression analysis of allergic rhinoconjunctivitis the final model included preeclampsia in addition to male gender and maternal asthma (Table 3). Specifically, severe preeclampsia gave 2.23 times higher odds for allergic rhinoconjunctivitis than no preeclampsia, adjusted for the other variables.

Adjusted for the same covariates as in final analysis, there was a trend of an increasing effect of preeclampsia (none, mild/moderate, severe) on both high level allergic sensitization (OR = 1.88; 95% CI: (1.23, 2.86); LR-p = 0.003) and on allergic rhinoconjunctivitis (OR = 1.42; 95% CI: (1.07, 1.89); LR-p = 0.018).

Preeclampsia was not a significant risk factor for other outcomes of atopic disease in the final models.

Preeclampsia was not a risk factor for any outcomes of lung function in unadjusted or fully adjusted linear regression analyses. Table 4 shows the results of linear regression analyses of lung function variables according to maternal preeclampsia status.

**Table 4:** Summary of linear regression analyses of lung function in late childhood in 395 Norwegian children according to mother’s preeclampsia status

**Effects of maternal preeclampsia**							
	**Fully adjusted**^**a)**^						
	**n**	**Mild/moderate preeclampsia**			**Severe preeclampsia**		
**Outcome variable**		**b**	**95% CI**	**p**	**b**	**95% CI**	**p**
FEV1%	381	0.57	(-1.86, 2.99)	0.647	1.27	(-2.95, 5.49)	0.555
FVC%	381	-1.47	(-4.36, 1.42)	0.317	-2.24	(-7.28, 2.79)	0.381
FEV1/FVC	381	1.28	(-0.35, 2.92)	0.124	2.79	(-0.06, 5.64)	0.055

*Abbreviations:FEV1%* forced expiratory volume in first second predicted, *FVC%* Forced vital capacity predicted, *FEV1/FVC* Ratio of actual FEV1 over FVC, *n* number of participants, *b* regression coefficient, *CI* confidence interval,

^a)^Adjusted for gender, birth weight z-score adjusted for gestational age, being firstborn, Caesarean section, maternal age, maternal smoking during pregnancy, gestational age in weeks, maternal body mass index (kg/m^2^) and maternal asthma.

## Discussion

In the present study we found positive associations between severe maternal preeclampsia and both high level allergic sensitization and allergic rhinoconjunctivitis in adolescent offspring. This is to our knowledge showed for the first time. Preeclampsia was not associated with subsequent atopic dermatitis, asthma or alterations in lung function.

### Preeclampsia and atopic disease

Few other studies have evaluated preeclampsia as a possible risk factor for subsequent allergic sensitization or atopic disease in a long-term perspective. Keski-Nisula et al. found an association between maternal preeclampsia and severe atopy in children. However, in that study only women who underwent caesarean section were included, and a very high percentage of children were sensitized [13]. Nafstad et al. found a relation between uterus-related complications during pregnancy and allergic rhinoconjunctivitis and asthma, but not for children born after preeclamptic pregnancies [11]. To our knowledge, no other studies have evaluated the risk for allergic rhinoconjunctivitis after preeclamptic pregnancies.

A possible causal relation between preeclampsia and atopic disease in offspring could be linked to the inflammatory changes observed during preeclampsia. If the tendency for preeclampsia to skew the cytokine profile of the mother towards an increased Th1/Th2 ratio is reflected in the cytokine pattern of the offspring during pregnancy and early life, it could potentially protect the child from the development of Th2 driven atopic disease [6,22]. However, preeclampsia is a complex inflammatory condition characterised by a variety of pro-inflammatory cytokines beyond the Th1 type of cytokines [10,23]. Pro-inflammatory cytokines, chemokines and adhesion molecules appear to be increased in maternal circulation during preeclampsia [13], and could potentially initiate the development of immunological conditions in the foetus, such as atopic sensitization or diseases [6].

The association between preeclampsia and atopy could be due to shared genetic or environmental factors in pregnancy. Preeclampsia is more common in nulliparous [24] and pregnancies with a male foetus [25], and atopic disease is more common in first-born-children [26] and boys up to adolescence [27]. According to the hygiene hypothesis, the birth order effect on atopy may be explained by a reduced tendency for Th2 deviation due to greater exposure to pathogens from older siblings [28]. However, recent studies have demonstrated a birth order effect on cord blood IgE and food allergy in very early life, suggesting a prenatal origin of this effect [29,30]. Our analyses were controlled for birth order, suggesting that preeclampsia may be a risk factor for atopy in the offspring unrelated to birth order. However, as this is an observational study, the possibility of residual confounding cannot be excluded.

Finally, maternal conditions prior to pregnancy could increase the risk for both preeclampsia and atopic disease in offspring. Maternal asthma has been shown as a risk factor for preeclampsia [31]. To our knowledge, no studies have shown any association between maternal allergy and preeclampsia.

There was a trend of an increased risk of atopic disease in the child by an increasing severity of the maternal preeclampsia. Moreover, preeclampsia was not associated with low level allergic sensitization, asthma or atopic dermatitis. Low level allergic sensitization may be unspecific and less related to clinical atopic disease compared to higher levels of sensitization [32]. The pathophysiology of asthma and atopic dermatitis is more multifactorial than the specific allergy driven pathophysiology of rhinoconjunctivitis. Our results may therefore suggest that the association between severe preeclampsia and atopic disease in offspring is related to specific Th2-mediated mechanisms [29].

### Preeclampsia, asthma, wheezing and lung function

Some studies have shown an association between different complications of pregnancy and asthma in offspring, but preeclampsia was not shown to be a risk factor in these studies [12,33]. In a large population-based study using a questionnaire, an association between maternal preeclampsia and wheezing in the offspring was shown [34]. A possible explanation for this association could be that hypertension in pregnancy is related to fetal growth restriction and hence altered airway function [35]. Our results do not contradict this. Although we could not find any association between preeclampsia and asthma ever, current asthma or lung function in late childhood, we did not investigate wheezing disorders in the first years of life. However, the present study had a longer follow-up than in the studies mentioned above, and may therefore be better suited to study any long time effect of preeclampsia on asthma and lung function in late childhood.

One limitation of the study is the rather low rate of participation, especially in FU2. It is not known whether there was a difference in prevalence of asthma or atopy between those who consented and those who didn’t consent to overall follow-up. Especially for the outcomes of asthma, there was a rather low response rate which increases the risk of a type 2 error. Furthermore, children who participated in FU1 but not in FU2 had a higher BMI and more atopic dermatitis. This may have biased our results, as both overweight and atopic dermatitis may be associated with allergic sensitization and other atopic disease.

Another limitation may be that that allergic rhinoconjunctivitis, asthma and atopic dermatitis were defined only by questionnaire. However, allergic sensitization was found in 94.4% of children diagnosed with rhinoconjunctivitis, suggesting a high degree of diagnostic accuracy. Some children reported allergic rhinoconjunctivitis without having sensitization, but this is also seen in other studies and does not rule out allergic rhinoconjunctivitis [36]. Due to study design, allergic rhinoconjunctivitis and allergic sensitization were assessed at two different ages. However, this should not affect that independent associations were found between preeclampsia pregnancies and allergic rhinoconjunctivitis and allergic sensitization respectively.

In the multivariate analyses we included a set of variables possibly influencing the outcomes. The covariates gestational age, birthweight z-score and caesarean section could be considered as intermediate variables between preeclampsia and the outcomes, but may also be independent risk factors for subsequent allergy and atopic disease and were therefore included as covariates in the analyses. Given the lack of complete ascertainment of causal links, one cannot exclude the possibility of collider bias and therefore biased associations between exposures and outcomes [37].

The only data on family atopy available were on maternal and paternal asthma. Paternal asthma was not considered to be a possible confounder for the relationship between maternal preeclampsia and subsequent atopy, asthma or lung function in offspring and not included as a covariate.

## Conclusion

The results of this study suggest that severe maternal preeclampsia may be associated with allergic sensitization and allergic rhinoconjunctivitis in late childhood. This emphasizes the possible early origin of atopic disease, but larger studies are needed to further explore the role of preeclampsia in the development of atopic disease. No other significant associations between maternal preeclampsia and atopic dermatitis, asthma or lung function were found.

## Competing interests

The authors declare that they have no competing interests.

## Authors’ contributions

KKB performed the registration of data, controlled the database, arranged the blood samples for analyses, performed statistical analyses, wrote a draft and completed the manuscript. BO drafted the primary study, was the leader of the data collection, performed registration of data, and critically revised the manuscript. GEE contributed to the outline of the tables, supervised statistical analyses and critically revised the manuscript. KØ supervised all parts of the study, the drafting, registration of data, and analyses, and contributed significantly to the writing of the manuscript. All authors read and approved the final manuscript.

## Pre-publication history

The pre-publication history for this paper can be accessed here:

http://www.biomedcentral.com/1471-2431/14/101/prepub
